# Supplementary material for: Investigation of Direct Electron Transfer of Glucose Oxidase on a Graphene-CNT Composite Surface: A Molecular Dynamics Study Based on Electrochemical Experiments
Source: Nanomaterials (Basel). 2024 Jun 24;14(13):1073. doi: 10.3390/nano14131073 (PMC11243339; doi:10.3390/nano14131073)
Supplement: Supplementary file 1 [file nanomaterials-14-01073-s001.zip › nanomaterials-3069130-supplementary.pdf]

Supporting information for

Investigation of Direct Electron Transfer of Glucose Oxidase on a Graphene-CNT Composite Surface: A Molecular Dynamics Study Based on Electrochemical Experiments

Taeyoung Yoon [1,†], Wooboum Park [1,†], Juneseok You [2,*] and Sungsoo Na [1,*]

[1] Department of Mechanical Engineering, Korea University, Seoul 02841, Republic of Korea; tyler1111@korea.ac.kr (T.Y.); pwb2002@korea.ac.kr (W.P.)
[2] Department of Mechanical Engineering, Kumoh National Institute of Technology, Gumi 39177, Republic of Korea
* Correspondence: proko1@kumoh.ac.kr (J.Y.); nass@korea.ac.kr (S.N.)
† These authors contributed equally to this work.

.

Contents:

1. **Molecular characteristics evaluation**

    1.1 RMSD

    1.2 MM/PBSA

    1.3 Marcus theory of electron transfer

    1.4 Laviron's methods for electron transfer rate

2. **Supplementary Figures**

3. **Supplementary movies**

1. Molecular characteristics evaluation

1.1 RMSD

$$RMSD = \sqrt{\frac{1}{N}\sum_{i=1}^{N} {\delta_i}^2}\ , \qquad \text{(S1)}$$

where δ_i is the distance between atom i and reference trajectory of the N equivalent atoms.

1.2 MM/PBSA

Free energy calculation correlates to evaluation of binding affinity and it has been performed with GROMACS plugin g_mmpbsa.

$$\Delta G_{binding} = G_{Immobilized} - (G_{enzyme} + G_{surface}) \qquad \text{(S2)}$$

Here, $G_{Immobilized}$ is the total free energy of the enzyme and surface, G_A and G_B denote enzyme and surface's total free energies in solvent, respectively. The total free energy was calculated by following equation.

$$G_{enzyme} = E_{MM} + G_{solvation} \qquad \text{(S3)}$$

where $G_{monomer}$ indicates enzyme monomer and graphene congeners sheet, E_{MM} is the average molecular mechanics (MM) potential energy versus time, which comprises bond, bond angle, dihedral, and non-bonded energies, and $G_{solvation}$ represents the solvation free energy for the entire MD simulation. Also, by using the MM/PBSA method, the solvation free energy was computed as follows.

$$G_{solvation} = G_{polar} + G_{nonpolar} \qquad \text{(S4)}$$

Here, G_{polar} and $G_{nonpolar}$ are distinguished by electrostatic effects on the solvation free energy. G_{polar} was estimated using the Poisson–Boltzmann equation, whereas $\boldsymbol{G_{nonpolar}}$ was computed using the following equation

$$G_{nonpolar} = G_{cavity} + G_{vdW} \qquad \text{(S5)}$$

where G_{cavity} is work done by the solute to create a cavity in the solvent and depends on the shape and geometry of the solute. G_{vdW} is the attractive van der Waals energy between solvent and solute. These terms can be estimated using a variety of models. In this paper, we calculated solvent accessible surface area (SASA) as follows.

$G_{nonpolar} = \gamma A + b$ *(S6)*

where Υ = 0.0226778 $kJ mol^{-1} \dot{A}^{-2}$ and b = 3.84928 kJ mol^{-1}.

1.3 Marcus theory of electron transfer

Interfacial DET between GOx and surface can only occur if the GOx active site is close enough. The rate constant for ET between a weakly coupled electron donor A and acceptor B in solution is described by the Marcus semi-classical theory as follows.

$$K_{ET} = \frac{4\pi^2 {H_{AB}}^2}{h\sqrt{4\pi\lambda K_B T}} exp\left[\frac{-(\Delta G+\lambda)^2}{4\lambda K_B T}\right] \quad \text{(S7)}$$

where h (Planck's constant), R (ideal gas constant), and K_B (Boltzmann's constant). H_{AB} represents the electron coupling between A and B, T is the temperature, ΔG is the free energy difference for ET, and λ is the reorganization energy. Here, electron coupling is mainly determined by H_{AB} as described below

$${H_{AB}}^2 = ({H_{AB}}^0)^2 e^{-\beta(r_{DA}-r_0)} \quad \text{(S8)}$$

where ${H_{AB}}^0$ is the electronic coupling at the vdW distance (r_0) and the tunnel parameter β reflects the efficiency of ET for the enzyme and it depends on the enzyme structure. Crespilho et al. derived the distance dependence of the ET rate constant for an active site that comprises of glucose oxidase and the electrode surface. [1] They simplified the equation by assuming the same β for different electrode materials and suggested a ratio for the ET rate of various created enzyme-electrode models. Thus, the minimum distance (*d*) between the center coordinate of the active site and surface can determine the ET rate.

In enzymes, an ET tunnel is formed through a maximum distance of ~1.4 nm. Absorption of the enzyme occurred within an adequate range of *d,* confirming that the distance was less than 1.4 nm. Consequently, the comparison of the ET rates can be achieved using Eq. (S5) by taking logs on both sides of Eq. (S4) and assuming the same β for the enzymes:

$$ln\left[\frac{K_{ET}\ (at\ mutated\ enzyme\ )}{K_{ET}\ (at\ wild\ type\ enzyme)}\right] = \frac{d_{MUT}}{d_{WT}} \quad (S9)$$

By substituting the ET rates calculated from Eq. (S5) into the simulated models,

1.4 Laviron's methods for electron transfer rate

Laviron's method was employed to determine the electron transfer (ET) rate constant of GOx adsorbed on the electrodes.[2] This method relies on the Tafel approximation of the Butler-Volmer equation, with the only necessary experimental data being the surface overpotentials as a function of current density. When the surface overpotential is sufficiently high, the general Butler-Volmer equation simplifies to the Tafel expression: ($i \approx -i_0 \exp\left(-\frac{\alpha F}{RT}\eta_s\right)$), where i is the current density, i_0 is the exchange current density, α is the transfer coefficient, and η_s is the surface overpotential. This simplified equation enables the evaluation of the transfer coefficient from the slope of the surface overpotential versus current density plot. Figure 8 displays the CV curves at scan rates ranging from 10 mV to 120 mV, with an inset graph illustrating the linear relationship between current density and overpotentials. The graph shows linearity in regions with high surface overpotentials. The transfer coefficient, α was determined from the slope of this linear part by using the following equation:[3, 4]

$$slope = -\frac{2.303RT}{\alpha nF} \quad (S10)$$

where R is the universal gas constant, T is the temperature in Kelvin, and n and F have their usual meanings as denoted in Eq. (S10). The transfer coefficient, α, had values of 0.516, 0.537, and 0.535 for CNT-GOx, Graphene-GOx and CNT/graphene-GOx respectively. K_{ET} can be determined by using Eq. (S11), where v is the x intercept of the linear voltage vs. log v plot and ΔE_p is the separation of the redox peak potentials.

$$logK_{ET} = \alpha\ log(1-\alpha) + (1+\alpha)log\alpha - log\left(\frac{RT}{nFv}\right) - \frac{\alpha(1-\alpha)nF\Delta E_p}{2.3RT} \quad (S11)$$

2. Supplementary Figures

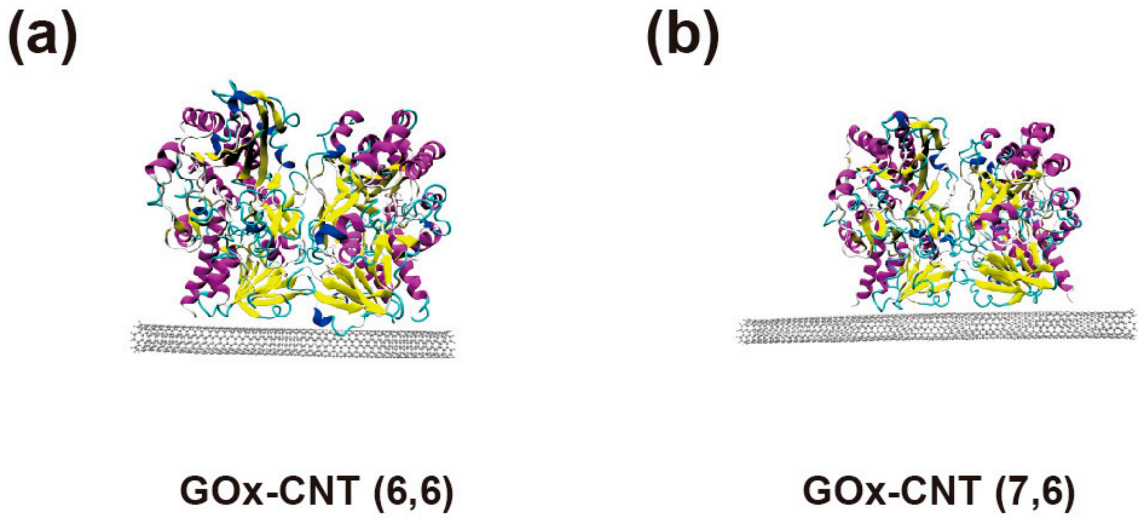

Figure S1. Visualization of the glucose oxidase (GOx) enzyme immobilized on (a) metallic carbon nanotubes (CNT) (6,6) and (b) semiconducting CNT (7,6).

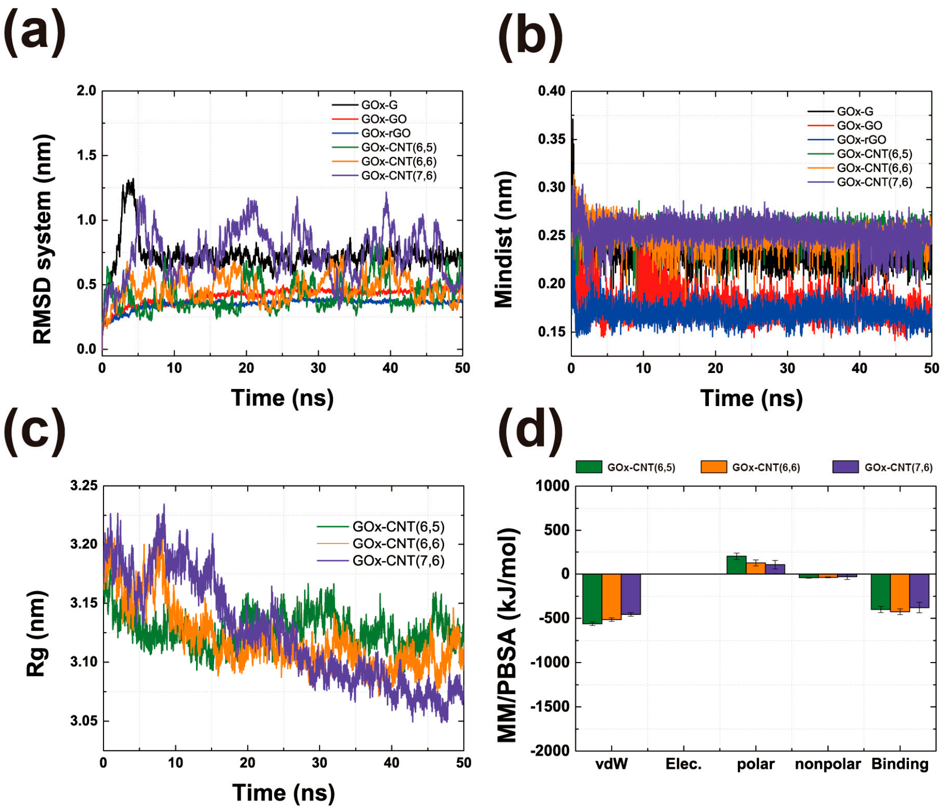

Figure S2. MD simulation results. (a) RMSD values and (b) Minimum distance between enzyme and surfaces over 50 ns MD trajectories. (c) Radius of gyration of MD trajectories of CNTs at different chiralities. (d) Binding energy calculation of CNT and enzyme depending on different chiralities.

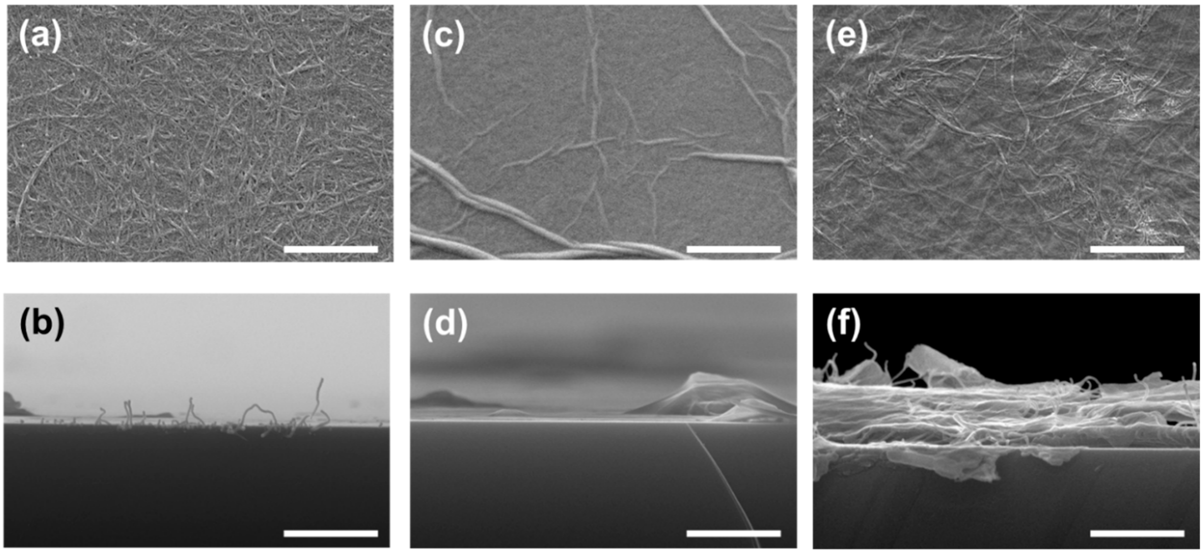

Figure S3. Top and side SEM view of CNTs (a, b), graphene (c, d), and graphene + CNT composite (e, f). The scale bar for (b) is 5 μm and it is 1 μm for the rest of the images.

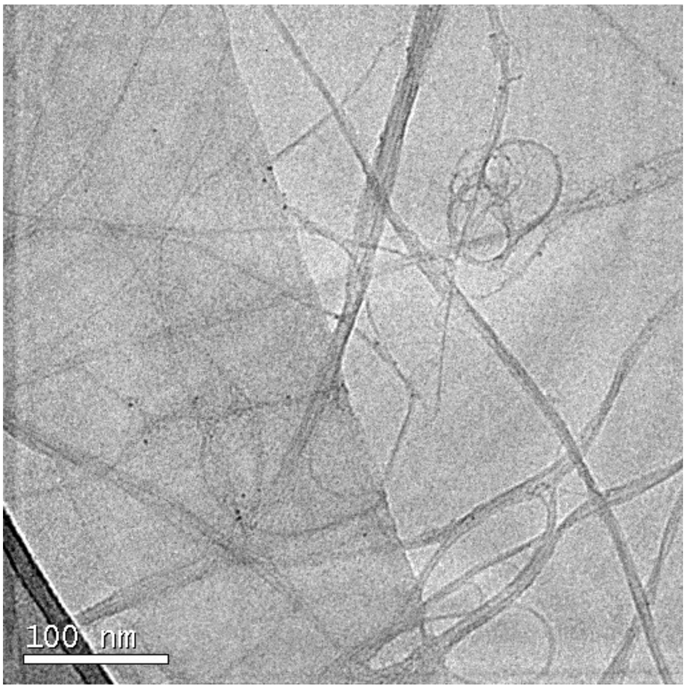

Figure S4. Transmission electron microscopy image of the graphene and CNT mixture.

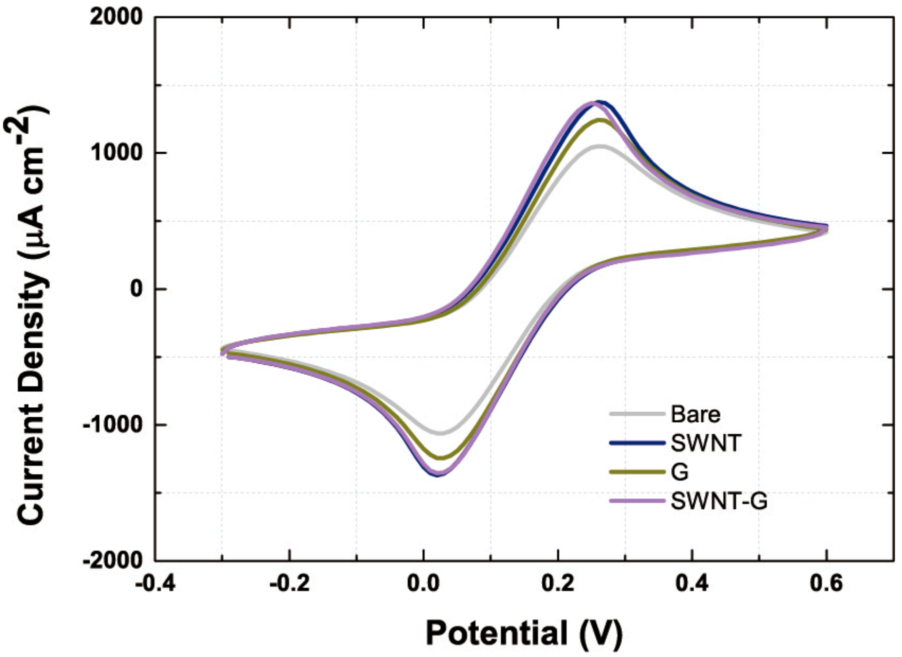

Figure S5. Cyclic voltammetry (CV) curves of $Fe(CN)_6^{-4}$ reduction without enzyme immobilization.

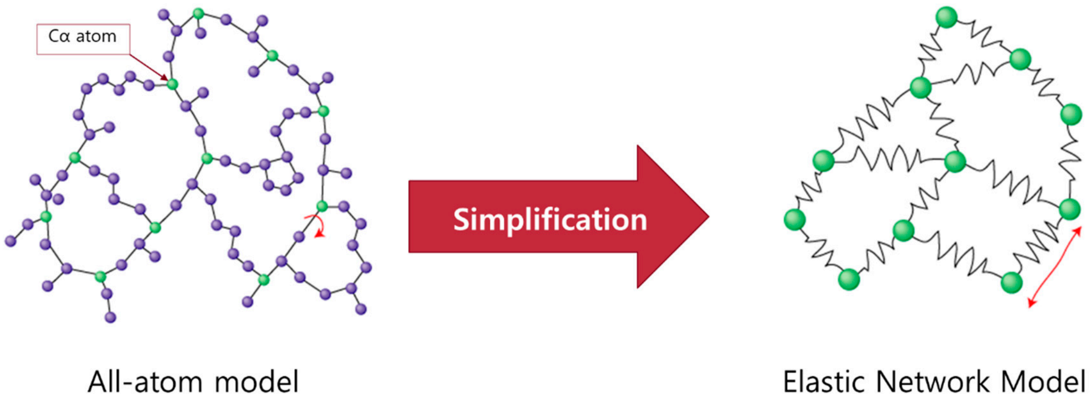

Figure S6. Schematics of elastic network model for protein analysis

SUPPORTING MOVIES

Supporting Movies S1-S3: Animation GIF files show mode 1-3 of GOx in bulk solution.

Supporting Movies S4-S6: Animation GIF files show mode 1-3 of GOx immobilized on pristine graphene.

Supporting Movies S7-S9: Animation GIF files show mode 1-3 of GOx immobilized on CNT(6,5)

3. References

[1] M.V. Martins, A.R. Pereira, R.A. Luz, R.M. Iost, F.N. Crespilho, Evidence of short-range electron transfer of a redox enzyme on graphene oxide electrodes, Physical Chemistry Chemical Physics, 16 (2014) 17426-17436.

[2] E. Laviron, General expression of the linear potential sweep voltammogram in the case of diffusionless electrochemical systems, Journal of Electroanalytical Chemistry and Interfacial Electrochemistry, 101 (1979) 19-28.

[3] P. Allen, A. Hickling, Electrochemistry of sulphur. Part 1.—Overpotential in the discharge of the sulphide ion, Transactions of the Faraday Society, 53 (1957) 1626-1635.

[4] A.J. Bard, L.R. Faulkner, Fundamentals and applications, Electrochemical Methods, 2 (2001) 482.
