# Supplementary figures and images for: Investigation of Direct Electron Transfer of Glucose Oxidase on a Graphene-CNT Composite Surface: A Molecular Dynamics Study Based on Electrochemical Experiments
Source: Nanomaterials (Basel). 2024 Jun 24;14(13):1073. doi: 10.3390/nano14131073 (PMC11243339; doi:10.3390/nano14131073)

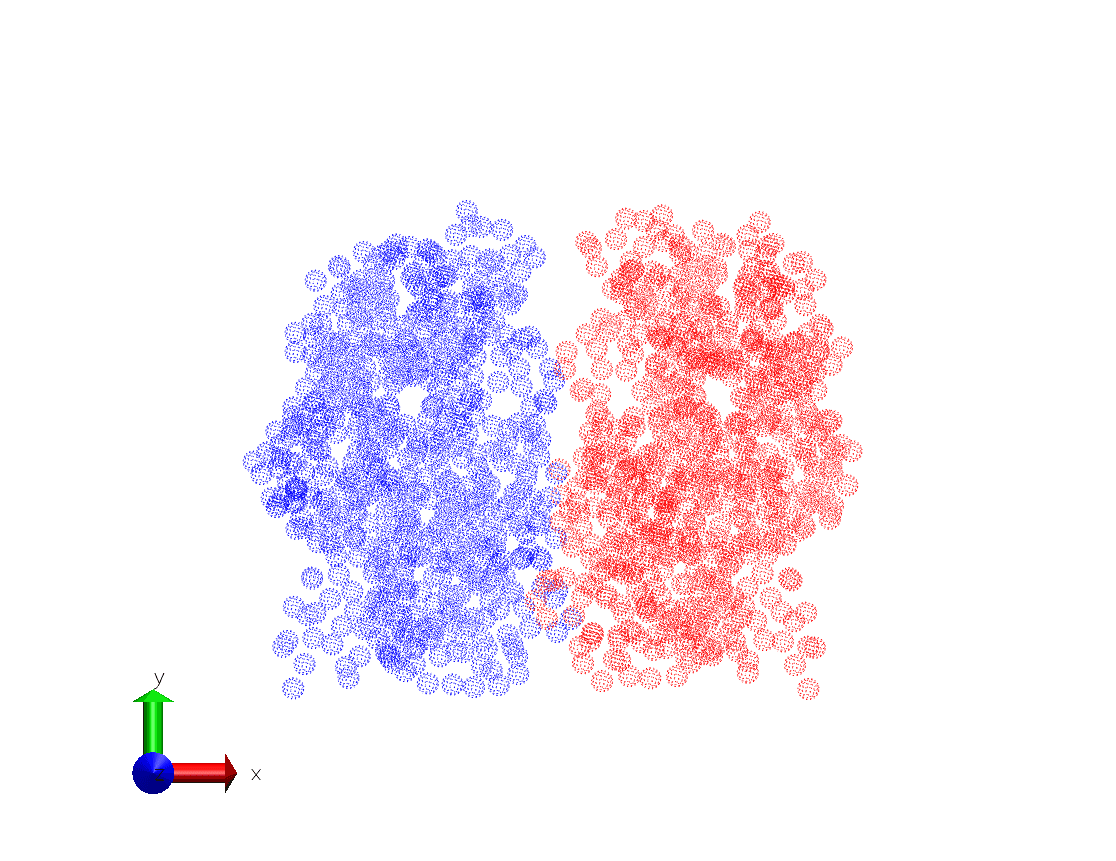

Supplement: Supplementary file 1 [file nanomaterials-14-01073-s001.zip › Movie S1_GOx_mode1.gif]

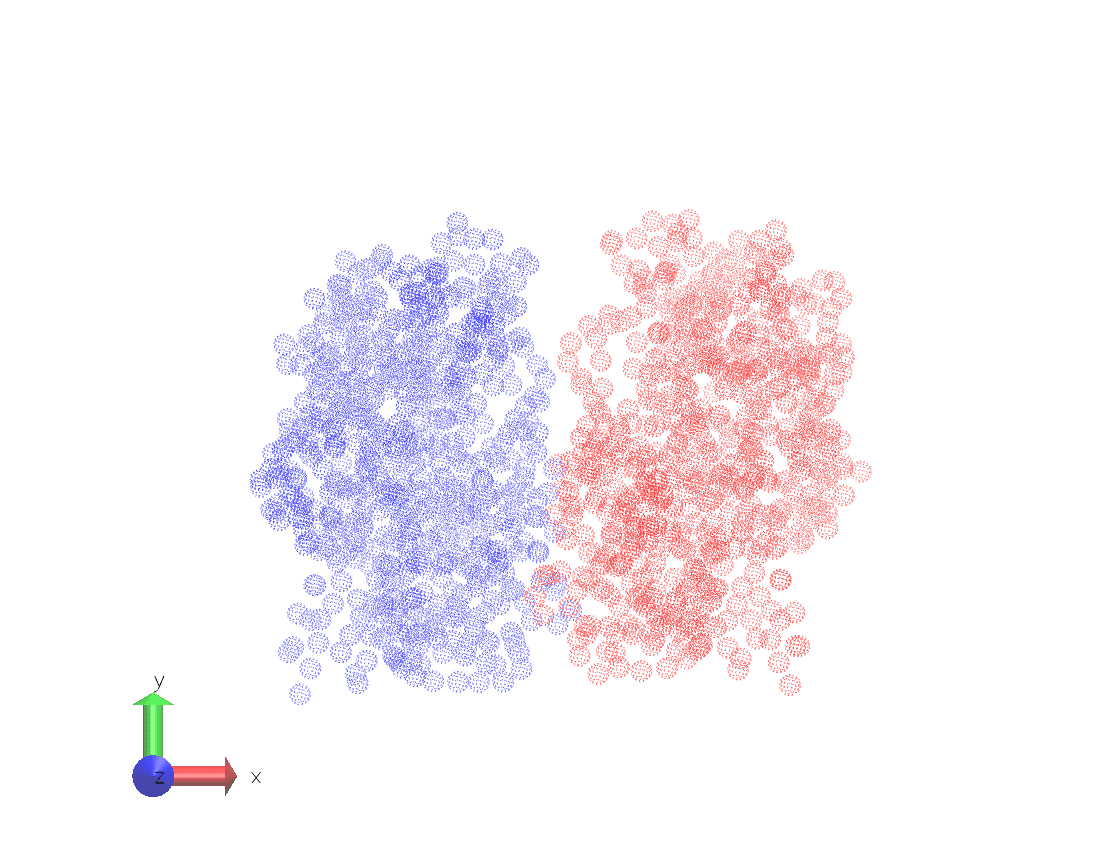

Supplement: Supplementary file 1 [file nanomaterials-14-01073-s001.zip › Movie S2_GOx_mode2.gif]

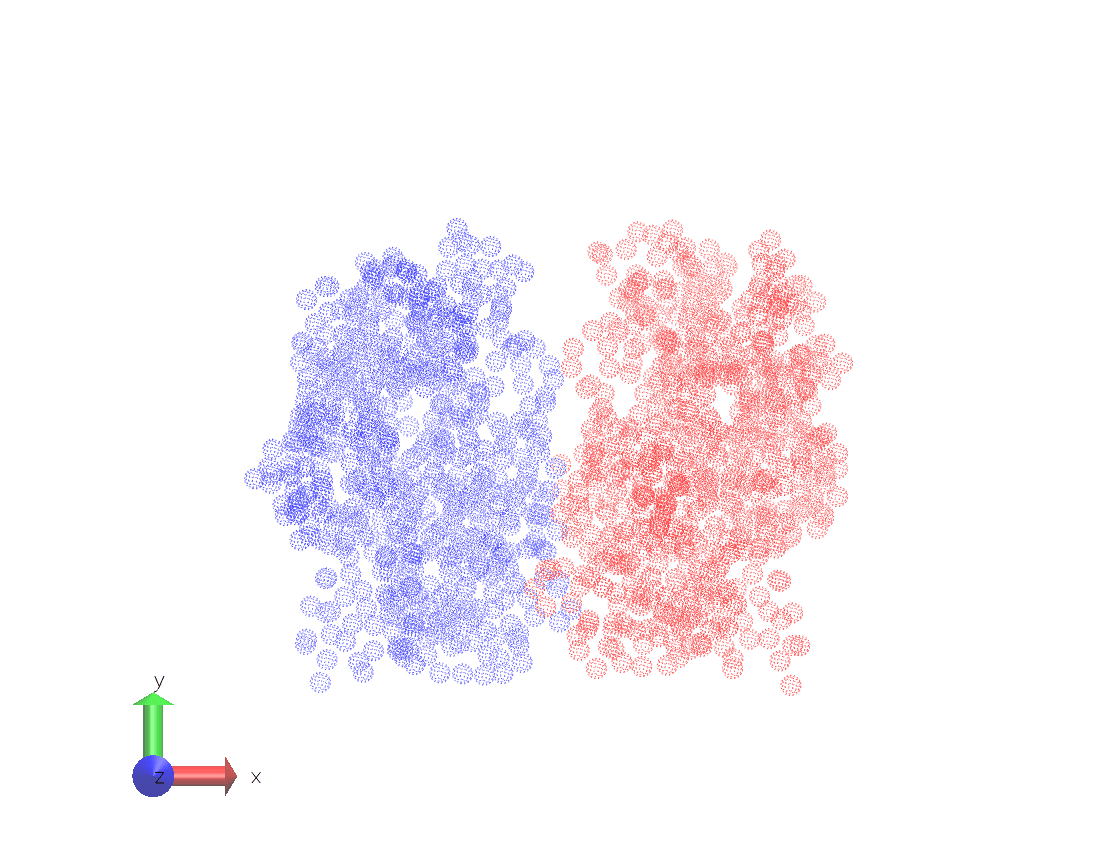

Supplement: Supplementary file 1 [file nanomaterials-14-01073-s001.zip › Movie S3_GOx_mode3.gif]

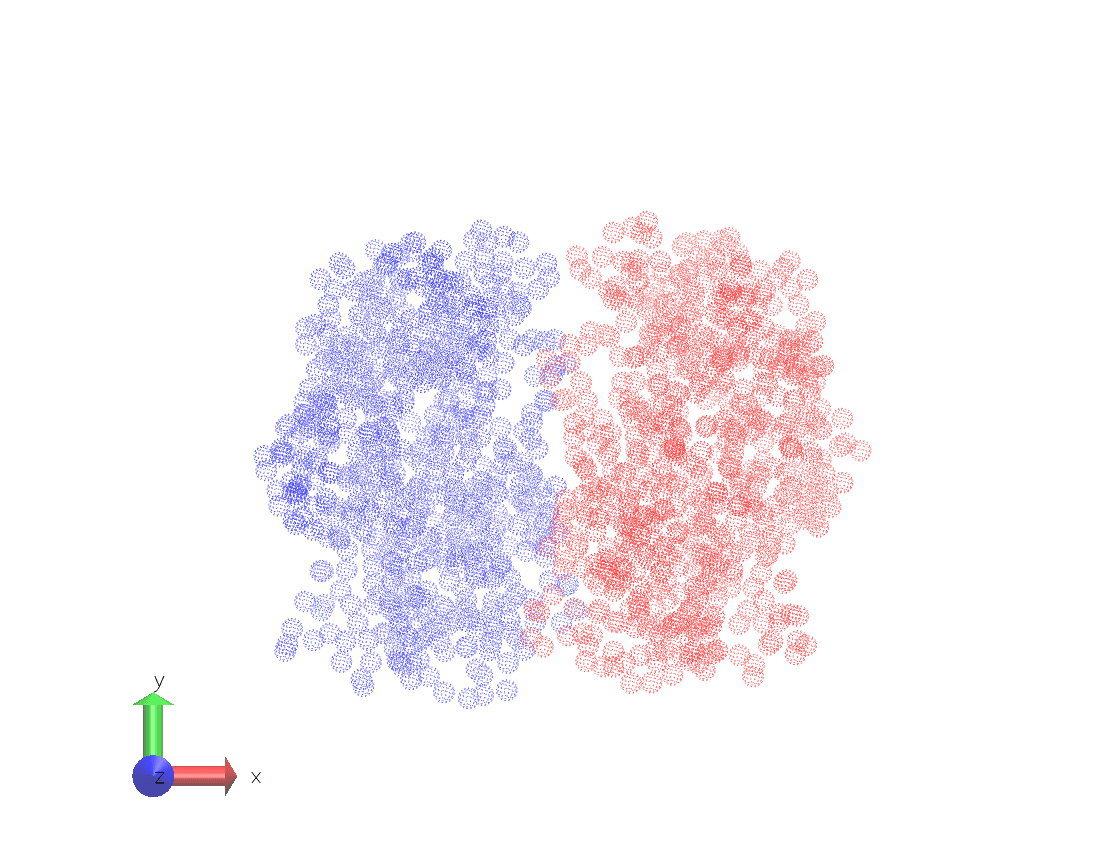

Supplement: Supplementary file 1 [file nanomaterials-14-01073-s001.zip › Movie S4_GOx_G_mode1.gif]

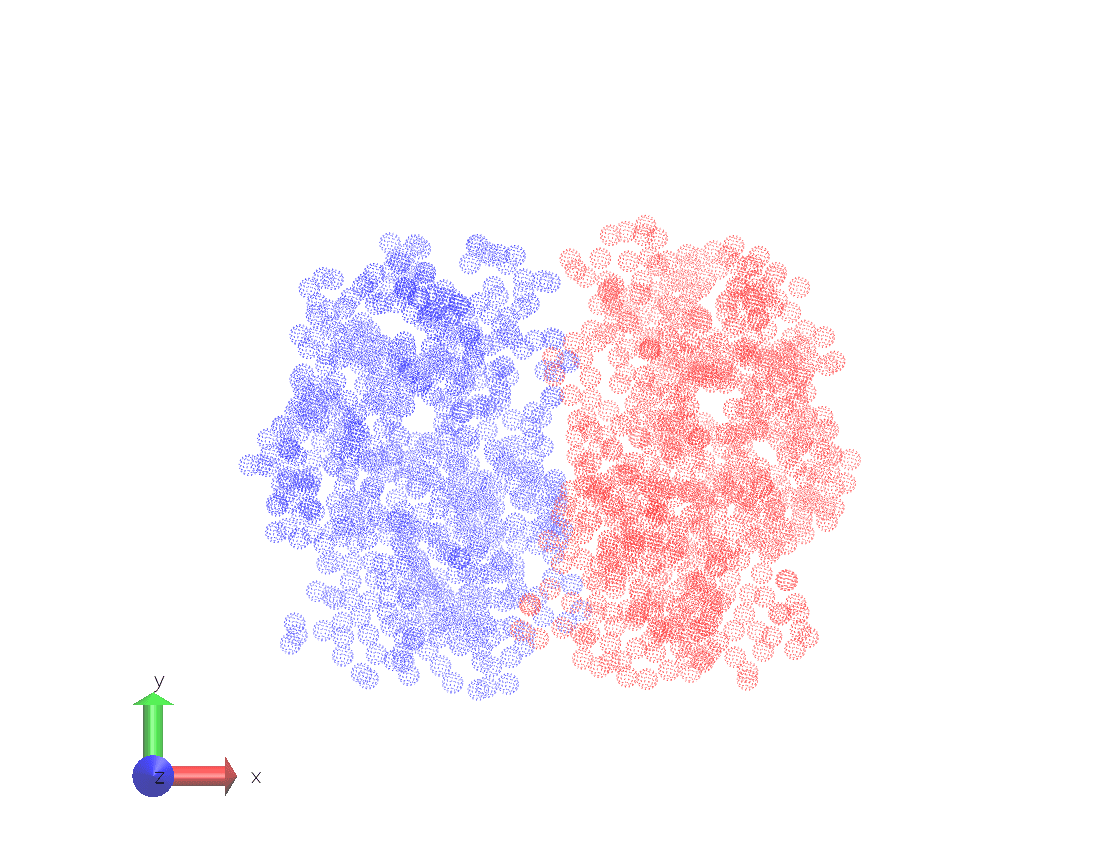

Supplement: Supplementary file 1 [file nanomaterials-14-01073-s001.zip › Movie S5_GOx_G_mode2.gif]

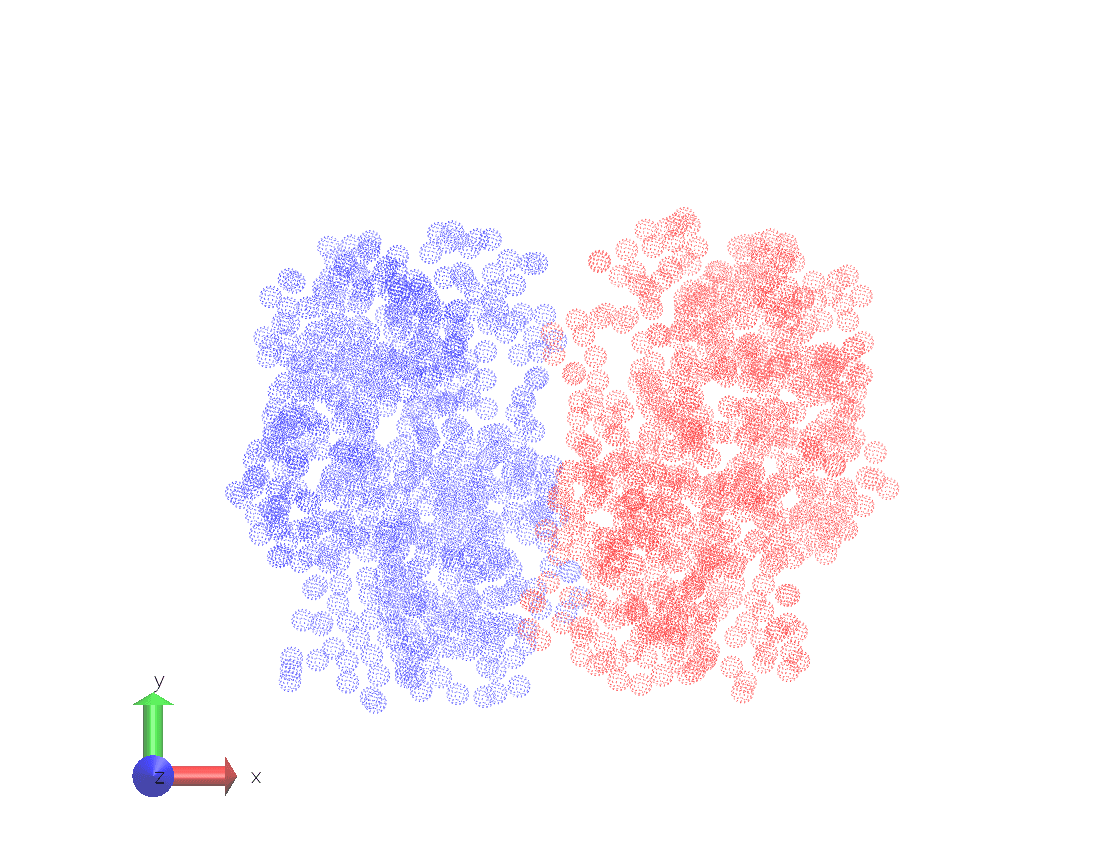

Supplement: Supplementary file 1 [file nanomaterials-14-01073-s001.zip › Movie S6_GOx_G_mode3.gif]

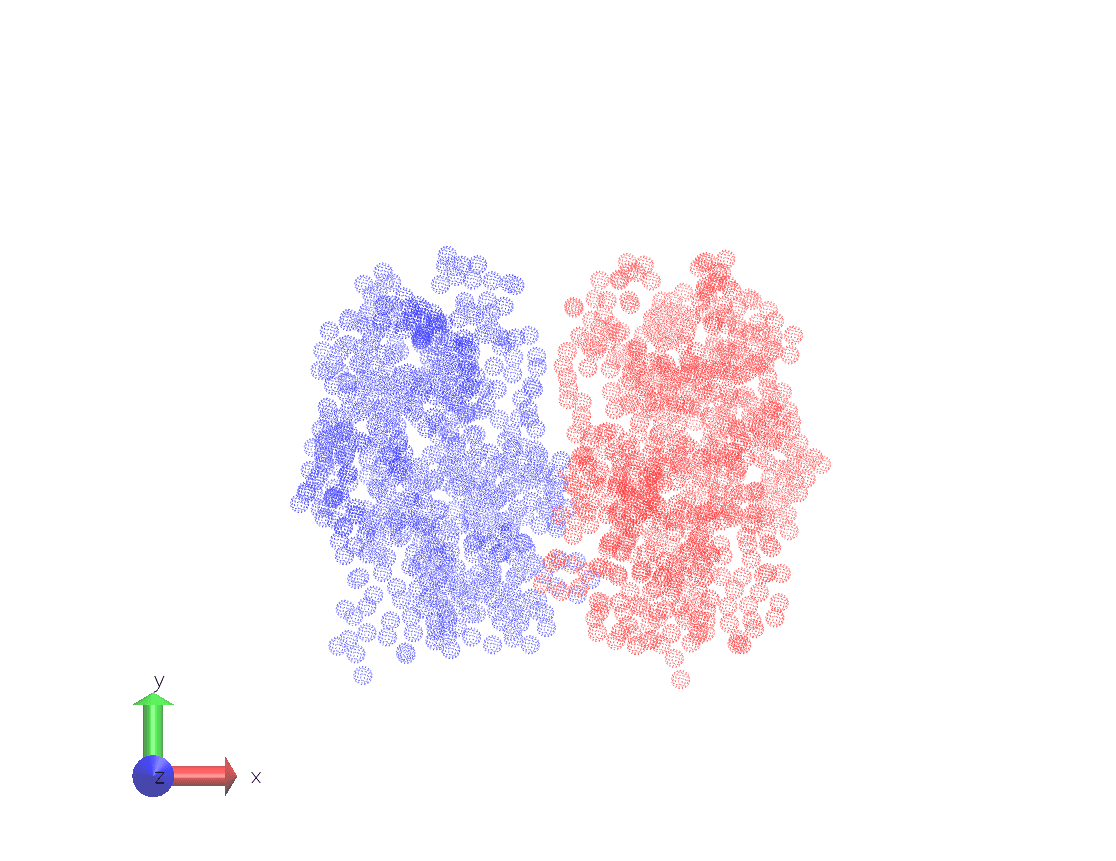

Supplement: Supplementary file 1 [file nanomaterials-14-01073-s001.zip › Movie S7_GOx_CNT(6,5)_mode1.gif]

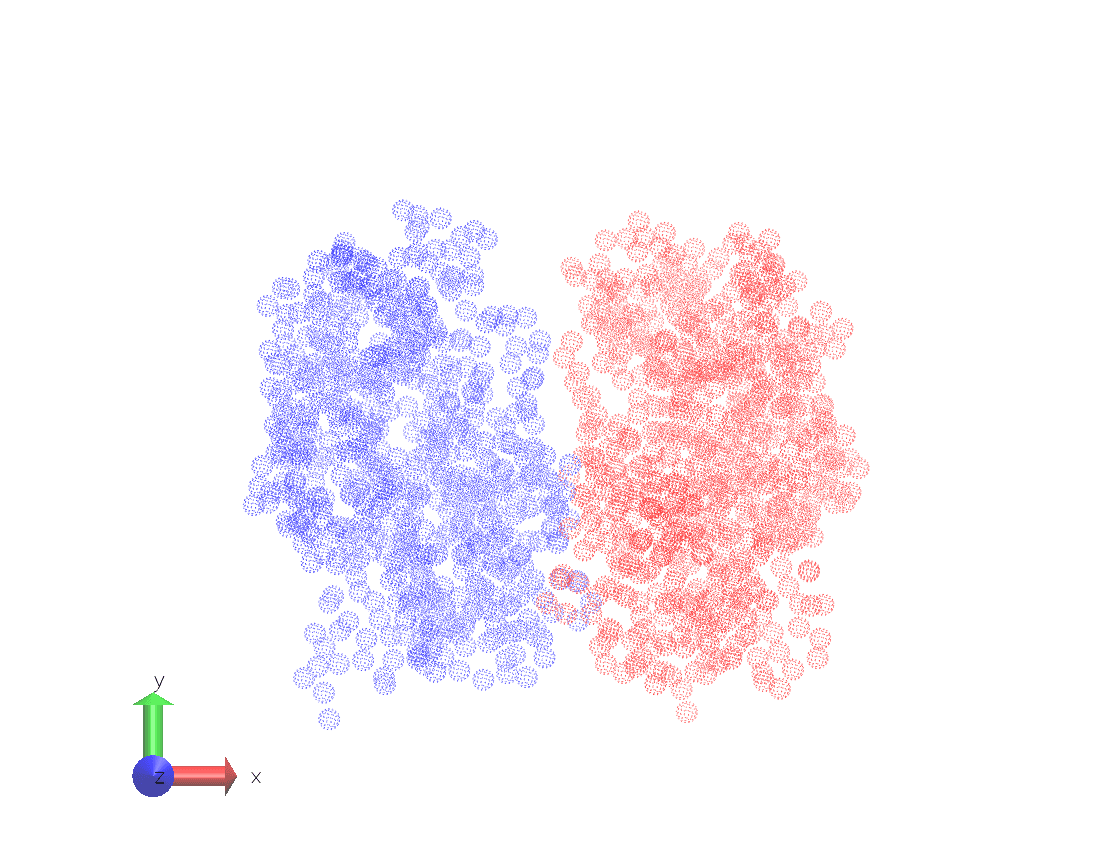

Supplement: Supplementary file 1 [file nanomaterials-14-01073-s001.zip › Movie S8_GOx_CNT(6,5)_mode2.gif]

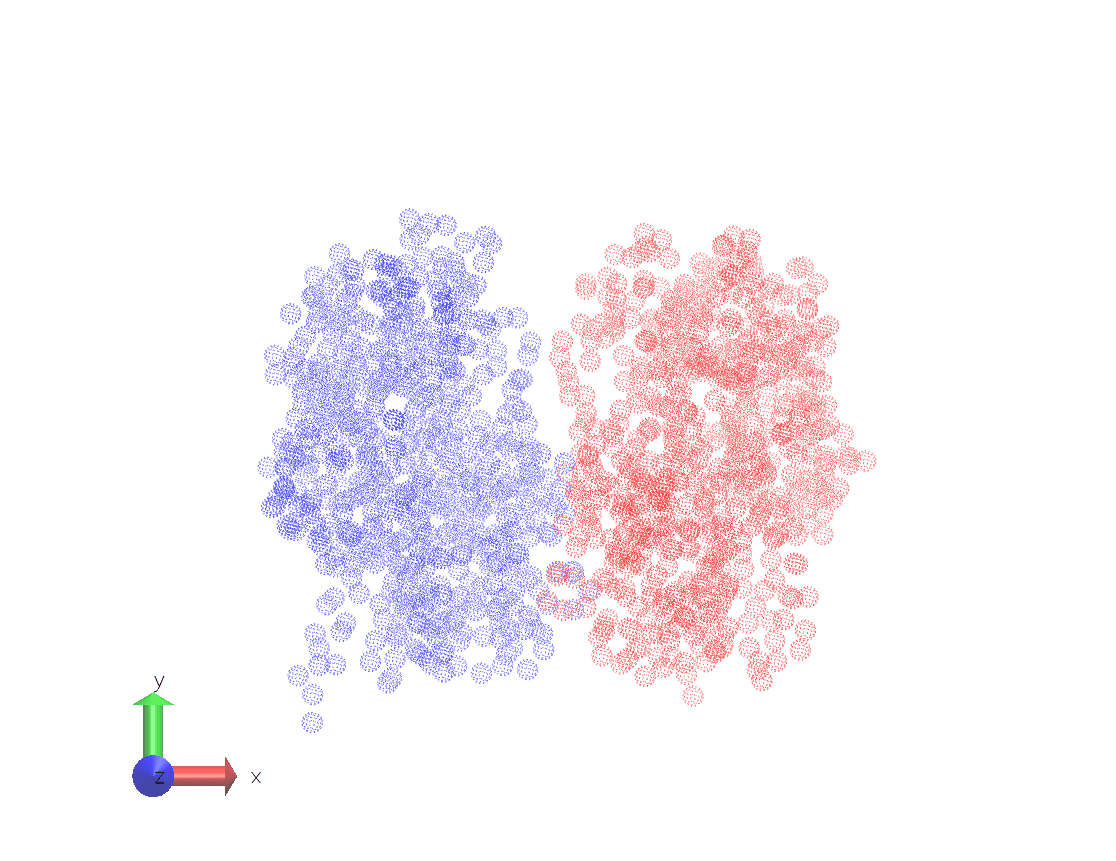

Supplement: Supplementary file 1 [file nanomaterials-14-01073-s001.zip › Movie S9_GOx_CNT(6,5)_mode3.gif]
